# Combined Effect of Cold Atmospheric Plasma and Curcumin in Melanoma Cancer

**DOI:** 10.1155/2021/1969863

**Published:** 2021-11-16

**Authors:** Zahra Yazdani, Pooyan Mehrabanjoubani, Alireza Rafiei, Pourya Biparva, Mostafa Kardan

**Affiliations:** ^1^Department of Basic Sciences, Sari Agricultural Sciences and Natural Resources University, Sari, Iran; ^2^Department of Immunology, Molecular and Cell Biology Research Center, School of Medicine, Mazandaran University of Medical Sciences, Sari, Iran

## Abstract

Curcumin (CUR) has interesting properties to cure cancer. Cold atmospheric plasma (CAP) is also an emerging biomedical technique that has great potential for cancer treatment. Therefore, the combined effect of CAP and CUR on inducing cytotoxicity and apoptosis of melanoma cancer cells might be promising. Here, we investigated the combined effects of CAP and CUR on cytotoxicity and apoptosis in B16-F10 melanoma cancer cells compared to L929 normal cells using MTT method, acridine orange/ethidium bromide fluorescence microscopic assay, and Annexin V/PI flow cytometry. In addition, the activation of apoptosis pathways was evaluated using BCL2, BAX, and Caspase-3 (CASP3) gene expression and ratio of BAX to BCL2 (BAX/BCL2). Finally, in silico study was performed to suggest the molecular mechanism of this combination therapy on melanoma cancer. Results showed that although combination therapy with CUR and CAP has cytotoxic and apoptotic effects on cancer cells, it did not improve apoptosis rate in melanoma B16-F10 cancer cells compared to monotherapy with CAP or CUR. In addition, evaluation of gene expression in cancer cell line confirmed that CUR and CAP concomitant treatment did not enhance the expression of apoptotic genes. In silico analysis of docked model suggested that CUR blocks aquaporin- (AQP-) 1 channel and prevents penetration of CAP-induced ROS into the cells. In conclusion, combination therapy with CAP and CUR does not improve the anticancer effect of each alone.

## 1. Introduction

The incidence of melanoma skin cancers has been increasing over the past decades so the World Health Organization (WHO) reported 132,000 confirmed cases of melanoma skin cancers to occur globally each year (https://www.who.int/uv/faq/skincancer/en/index1.html). Melanoma cells become “bullet proof” against a variety of clinical managements, including chemotherapy, radiotherapy, and immunotherapy by exploiting their intrinsic resistance to apoptosis, reprogramming their proliferation and survival pathways during melanoma progression [1].

Cold atmospheric plasma (CAP) has recently emerged as a novel tool in biomedical applications [2]. It is an ionized gas composed of reactive oxygen (ROS) and reactive nitrogen (RNS) species and an optical emission in the UV range [3]. The significant rise of the CAP-induced reactive species induces a selective cell death in multiple cancer cell lines, including melanoma [4, 5], breast [6, 7], glioblastoma [8], leukemia [9], and head and neck [10] cancer in vitro, and decreases the size of solid tumors in vivo [11–15]. CAP kills cancer cells and does not have a catastrophic effect on normal cells [3]. The selective anticancer mechanism of this modality is due to a significant different level of ROS between cancer and normal cells [3]. To explain this difference, two models have been proposed. The first model proposed that cancer cells have a stronger metabolism and a higher baseline ROS level than normal cells. When CAP-induced ROS is applied to cells, total intracellular ROS in cancer cells easily exceeds the threshold, but this does not occur in normal cells. Therefore, cancer cells experience more apoptosis in comparison with normal cells [3, 16, 17]. The second model is based on the presence of aquaporin (AQP) channels. These channels are transmembrane proteins for the permeation of reactive oxygen species, including H_2_O_2_, NO_3_^−^, and NO into the cells [3]. The cancer cells express more AQPs in comparison with normal cells. Thus, CAP causes ROS to penetrate more and more into cancer cells and thus causes more apoptosis in tumor cells than in normal cells [18, 19].

Apart from the ameliorative activity of CAP, some natural compounds have been identified with anticancer properties [20]. Curcumin (CUR) is a natural compound that has potentially anticancer properties [21]. It is a yellow pigment from Curcuma longa. Its anticancer activity was reported in various types of cancers such as ovarian, lung, breast, and melanoma [22–27]. However, the mechanism of anticancer action of CUR is not fully understood; some studies showed it kills cancer cells by induction of apoptosis [26, 27].

It can be assumed that the combined use of CAP with another anticancer agent may have a synergistic effect in the treatment of cancer [15, 28–31]. Therefore, the aim of this study was to evaluate the effect of CAP and CUR combination therapy on apoptosis of B16-F10 melanoma cell line in comparison with normal L929 fibroblast cell line. Also, in silico study investigated their potential molecular interactions.

## 2. Materials and Methods

### 2.1. Chemicals and Reagents

MTT (3-(4, 5-dimethylthiazol-2-yl)-2, 5-diphenyltetrazolium bromide) and dimethyl sulphoxide were purchased from Sigma-Aldrich, USA. RPMI-1640 and penicillin-streptomycin were obtained from Biowest, Germany. Fetal bovine serum and trypsin were purchased from bioMerieux, France, and Gibco, USA. Ethidium bromide and acridine orange were purchased from Merck, Germany. Cell culture plates and flasks were purchased from SPL, Korea, and the micro tubes were purchased from Ratiolab, Germany. All primers were synthetized by Metabion, Germany. The used kits in the study include RNA extraction kit (FAVORGEN, Taiwan), cDNA synthesis kit (Addbio, Korea), and FITC Annexin V Apoptosis Detection Kit (Bioscience, USA).

### 2.2. Cold Atmospheric Plasma Device

The experiments were performed with a plasma jet, which is generated from an argon flow. The distance between the target cells and plasma source nozzle was 3 centimeters. Technical details of the device were described previously [32–34].

### 2.3. Cell Culture

B16-F10 and L929 cell lines were cultured in RPMI-1640 containing 10% (*v*/*v*) fetal bovine serum, 100 U/ml penicillin, and 100 mg/ml streptomycin. The cells were maintained at 37°C in a humidified incubator containing 5% (*v*/*v*) CO_2_ [20]. When the cells reached 70% confluence, they were harvested using 0.25% trypsin and used for 2.4-2.6 stages. The number of cell passages was 0-2 times for each test.

### 2.4. Cell Survival Assay

MTT assay was used to measure cytotoxicity effect of each treatment [35]. Firstly, 8 × 10^3^ cells/well of B16-F10 and 1 × 10^4^ cells/well of L929 were seeded in 96-well cell culture plates in triplicate. To treat by CUR, after initial 24 h incubation, the cells were cultivated with fresh media containing different concentrations (0-100 *μ*M) of CUR (culture medium and 0.5% (*v*/*v*) DMSO was used as solvent). To evaluate the therapeutic effect of CAP, the cells were exposed to CAP in various times (untreated, 20, 30, 40, 50, and 60 seconds (s)) and then incubated for 24 or 48 h. To assess the combination effect of CUR and CAP, the cells were treated simultaneously with CUR and immediately with CAP. Overall, to evaluate the cytotoxic effect of CUR and CAP combined therapy, we used optimal dose of each treatment. Therefore, four experiment groups were considered as untreated that received no treatment, CAP treated, CUR treated, and combined CAP and CUR treated. The cells in each study group were then treated with 20 *μ*l of MTT reagent (5 mg/ml in sterile phosphate-buffered saline (PBS)) and incubated at 37°C for 4 h. Finally, the culture medium was removed and 200 *μ*l of DMSO was added to dissolve the formazan crystals. Optical density of each well was measured at 570 nm by a microplate reader (BioTek, Instruments Inc., Vermont, USA). The percentage of cell viability was calculated based on the optical density of the wells. IC_50_ was developed by an inhibition curve and recorded as the mean ± standard deviation of three independent experiments [20].

### 2.5. Acridine Orange/Ethidium Bromide (AO/EB) Staining for Apoptosis Detection

8 × 10^3^ cells/well of B16-F10 and 1 × 10^4^ cells/well of L929 were seeded in each well of the 96-well culture plate. After 24 hours of incubation, the cells were treated with combined CUR and CAP for 24 hours. Then, cells were washed twice with PBS and stained with 10 *μ*l of AO (50 *μ*g/ml) and 10 *μ*l of EB (50 *μ*g/ml). The cells were observed using a fluorescent microscope (Motic, China) in 470/40 nm at ×1000 magnification [20].

### 2.6. Flow Cytometric Analysis

Analysis of cell death was determined by staining the cells with an Annexin V/propidium iodide (PI). The cells were incubated for 24 hours in 6-well cell culture plates after treatment with CUR, CAP, or the combination of CAP and CUR. The cells were then harvested, washed with PBS, and suspended in Annexin V binding buffer. They were incubated for 15 min at room temperature. Subsequently, FITC-Annexin V reagent was added to each tube and the tubes were incubated for 10 min at room temperature. Then, the cells were stained by PI. Finally, the pattern of cell death was analyzed using FACSVerse (Partec, Germany).

### 2.7. qRT-PCR

Total RNA was extracted using the RNA extraction mini kit (FAVORGEN, Taiwan) according to the manufacturer recommendations. Then, qRT-PCR was performed by stem-loop TaqMan real-time PCR assay, using unique sequence index (USI) barcodes and probe described by Fattahi et al. [33, 36]. Gene amplification was carried out using a StepOnePlus™ real-time PCR system (Applied Biosystems, CA, USA), and the results were expressed as the fold change calculated using the 2^-*ΔΔ*Ct^ method relative to the control sample. GAPDH was used as a housekeeping gene to normalize gene expression. Primer sequences were designed by AlleleID 6.0 software and showed in Table 1. To amplify the genes, we used the following thermal profile: initial denaturation at 95°C for 5 minutes and then 40 repetitions at 95°C for 15 seconds and 60°C for 60 seconds.

### 2.8. Computational Modeling

CUR interaction with AQP-1 was investigated to study the effect of CUR on blocking ROS transportation into aquaporin channels. Then, this docking model was compared with the docking model of the acetazolamide interaction and AQP-1. Ligand structures of CUR and acetazolamide were obtained from the PubChem database and stored in SDF format. These structures were converted to PDB format using Open Babel GUI. Hydrogen atoms were added by Discovery Studio 4.5 and subjected to ligand preparation to generate possible conformations and PDBQT format using AutoDock tools 1.5.6. Protein structure of AQP-1 with the code 1H6I was obtained from the PDB database and stored in PDB format. Then, all the crystallographic water molecules were removed and hydrogen polar atoms were added using Discovery Studio 4.5. Finally, AutoDock tools 1.5.6 removed all the crystallographic water molecules and converted protein structure to PDBQT format. Protein-protein docking of the AQP-1 structure (as a receptor) with CUR and acetazolamide (as ligands) was performed by AutoDock Vina, and the results were shown in PDB format using Discovery Studio 4.5 software [37].

### 2.9. Statistical Analysis

Quantitative data were presented as mean ± SD or SE, appropriately. The Student *t*-test and one-way ANOVA were used to compare quantitative variables. In addition, Tukey post hoc test was used for comparison between groups.

## 3. Results

### 3.1. Effect of CUR on Cell Viability

**Figure 1:**
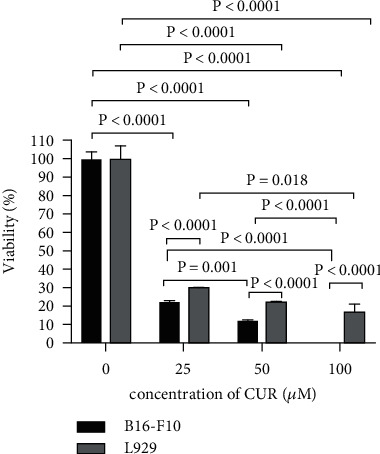
Effect of curcumin on the viability of B16-F10 and L929 cell lines. The cells were treated with different concentrations of curcumin for 24 h. Results are presented as mean ± SD. Statistical analysis was performed using a Student *t*-test and a one-way ANOVA test followed by Tukey's post hoc test for comparisons. CUR: curcumin.

To determine the CUR cytotoxicity, B16-F10 and L929 cell lines were treated with various concentrations of CUR for 24 h and viability of cells was measured using MTT assay. As Figure 1 shows, the viability of untreated cells (treated cells with solvent and no CUR) did not decrease. CUR decreased the cell variability in a dose-dependent manner. On the other hand, the cytotoxicity effect of CUR on B16-F10 tumor cells was significantly higher than that on L929 cells (*P* < 0.0001). IC_50_s of CUR on B16-F10 and L929 cells were 16 and 22 *μ*M, respectively. In this manner, three concentrations of 15, 20, and 25 (*μ*M) showed approximately the same results in the cytotoxicity assay. The concentration of 20 *μ*M which has the best cytotoxic effect on tumor cells with less side effect on the normal cells was used in subsequent experiments (Figure 1, Figures S1 and S2).

### 3.2. Effect of CAP on Cell Viability

**Figure 2:**
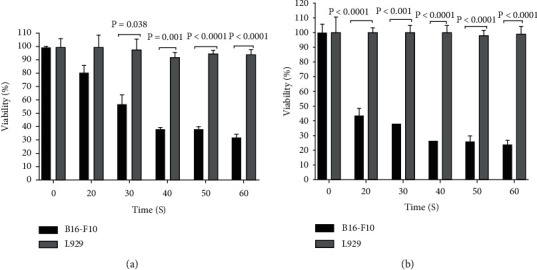
Effect of cold atmospheric plasma on the viability of B16-F10 and L929 cell lines. The cells were exposed by various times (0 to 60 seconds) of CAP and then incubated for (a) 24 h or (b) 48 h. Results are presented as mean ± SD. Statistical analysis was performed using a Student *t*-test and a one-way ANOVA test followed by Tukey's post hoc test for comparisons. CAP: cold atmospheric plasma.

Following 24 h incubation after CAP exposure, the survival of B16-F10 cells decreased significantly in a dose-dependent manner, while L929 cell viability did not change significantly (Figure 2(a)). Increasing in incubation time from 24 to 48 h caused a significant reduction in the viability of B16-F10 cells but not in L929 normal cells (Figure 2(b)). 40-second CAP exposure had the highest cytotoxic effect on B16-F10 cancer cells without significant detrimental effect on normal cells. Therefore, this time was considered as the appropriate CAP exposure time in subsequent experiments.

### 3.3. Effect of Combined CUR and CAP on Cell Viability

**Figure 3:**
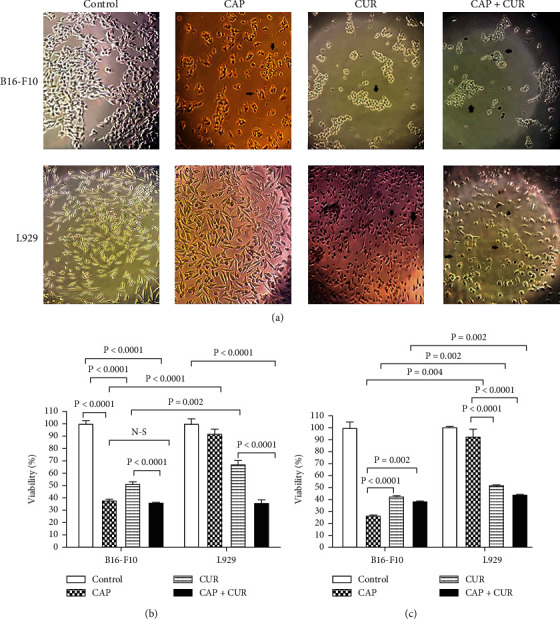
Cytotoxic effect of combination therapy with CAP and CUR on B16-F10 melanoma cancer and L929 normal cell lines. (a) Morphological alterations visualized under the light convert microscope. The black arrows showed rounded and wrinkled membrane cells. (b) Cell viability was shown using MTT test after 24 h treatment. (c) Cell viability was shown using MTT test after 48 h treatment. CAP: cold atmospheric plasma; CUR: curcumin; CAP+CUR: combination therapy of cold atmospheric plasma and curcumin; N-S: nonsignificant.

With regard to the cytotoxicity of CAP and CUR on the cancer cells, we decided to assess the cytotoxic effect of combination therapy of these modalities on the B16-F10 cancer and L929 normal cells. Microscopic images showed that all treatments had a significant effect on the morphology of the B16-F10 melanoma cells. As Figure 3 shows, these cells became rounded, and their membranes wrinkled. CAP did not alter the morphology of L929 cells compared to untreated cells, whereas CUR significantly altered the morphology of these cells. In other words, CUR rounds L929 cells and weakens their attachment to the bottom of the plate. Combination therapy of CUR and CAP caused more damage to the morphology of cancer cells than CUR alone. Comparison of the cell morphology of two cell lines showed CAP has selective toxicity on tumor cells. Interestingly, the combined CUR and CAP had a more catastrophic effect on the morphology of the B16-F10 cancer cells compared to normal L929 cells. Furthermore, to quantify cytotoxicity effect of each treatment on tumor cells, MTT assay was used. After 24 h and 48 h of treatment, all treatment modalities inhibited significantly the viability of B16-F10 cells when compared to the untreated control (*P* < 0.0001). The toxic effect of combination therapy of CUR and CAP on melanoma cells was not significantly higher than the monotherapy with CAP. In the periods of 24 h and 48 h, treatments of CAP and CUR showed the inhibitory effects on the viability of melanoma cells compared to untreated cells, but combination therapy had no more cytotoxicity effect on melanoma cancer cells than fibroblast normal cells.

### 3.4. Assessment of Apoptosis by AO/EB Staining

**Figure 4:**
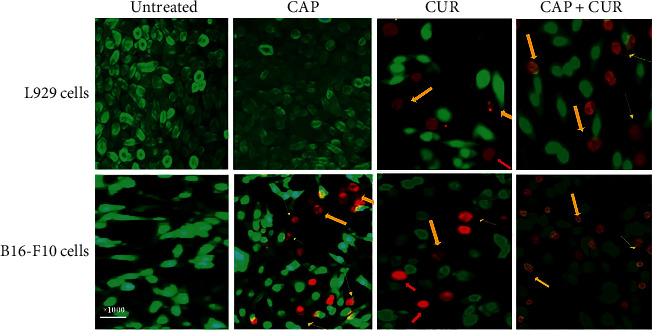
AO/EB staining of L929 normal fibroblast and B16-F10 melanoma cells. The cells were treated with CAP (40 seconds), CUR (20 *μ*M), or combination therapy with CUR and CAP. Live cells are green in color while apoptotic cells are indicated by yellow arrows and necrotic cells with red arrows. CAP: cold atmospheric plasma; CUR: curcumin; CAP+CUR: combination therapy of cold atmospheric plasma and curcumin.

AO/EB double fluorescent assay was performed to indicate cell apoptosis in B16-F10 and L929 cell lines. AO permeates all cells and represents the cells fluorescence green while EB stained dead cells, which shows the nuclei to be the red color. Therefore, the live cells appeared green color while the apoptotic cells illuminated by red nuclei and condensed or fragmented chromatin. Uniformly red cells indicate direct necrosis. The results showed CAP induces apoptosis in the B16-F10 cancer cells but has no toxic effect on L929 normal cells, while CUR alone or in combined therapy induces apoptosis and necrosis on both cancer and normal cells (Figure 4).

### 3.5. Detection of Death Pattern by Flow Cytometry

**Figure 5:**
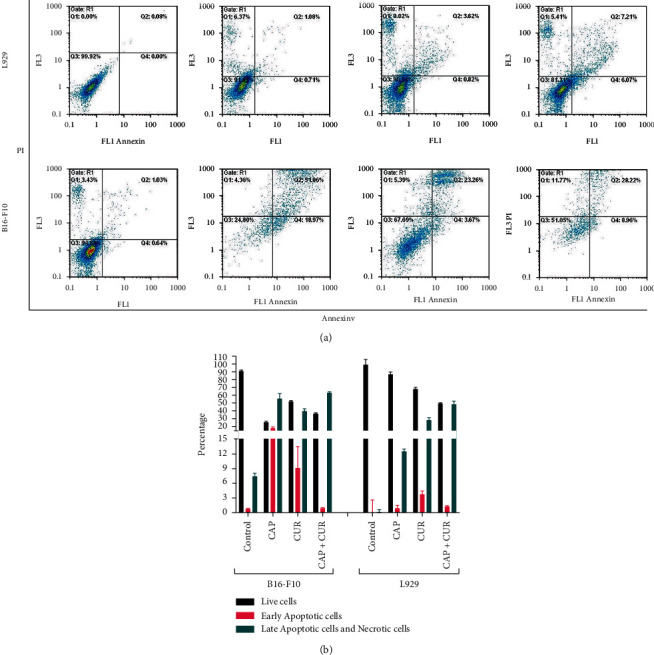
Flow cytometric chart of Annexin V/PI staining of L929 normal and B16-F10 cancer cell lines after harvesting by trypsinization. (a) Vital (Annexin V^−^PI^−^), early apoptosis (Annexin V^+^PI^−^), late apoptosis and necrosis (Annexin V^−^PI^+^), and necrosis (Annexin V^+^PI^+^). Dot blots are representative of a group of particles.

Annexin V/PI assay was used to quantify the death pattern in melanoma B16-F10 and normal L929 cells.  Figure 5 shows that CAP did not significantly induce apoptosis or necrosis in normal L929 cells, whereas the apoptosis rate was higher in tumor and normal cells when treated by CUR alone or in the combination therapy compared to untreated cells. On the other hand, all therapeutic approaches induced apoptosis in the B16-F10 tumor cells, and in addition, combination therapy increased necrosis compared to the CAP or CUR monotherapy.

### 3.6. Expression of Apoptosis-Related Genes

**Figure 6:**
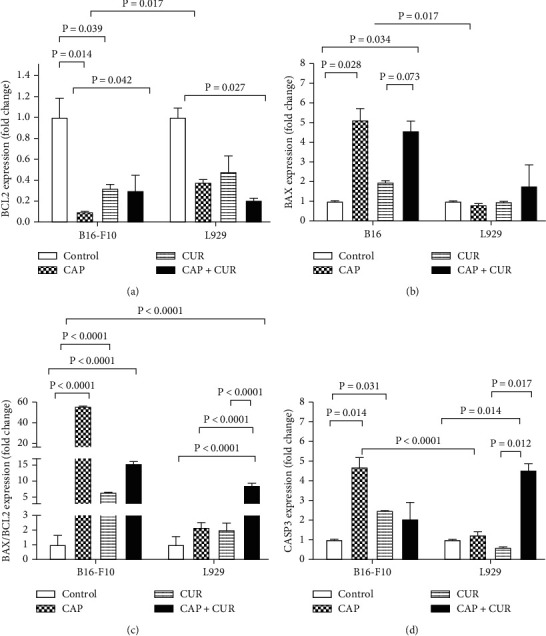
Expression of apoptotic genes after treatment of CAP, CUR, or combination therapy of CUR and CAP: (a) BCL2 gene, (b) BAX gene, (c) BAX/BCL2 ratio, and (d) CASP3 gene. Data are the mean ± SE of three independent experiments. Statistical analysis was performed using a Student *t*-test and a one-way ANOVA test followed by Tukey's post hoc test for comparisons. CAP: cold atmospheric plasma; CUR: curcumin; CAP+CUR: combination therapy of cold atmospheric plasma and curcumin.

The expression of apoptotic genes including BAX, BCL2, and CASP3 was evaluated in B16-F10 melanoma cancer and L929 cells by real-time PCR assay. As Figure 6 shows, the expression of all genes and the BAX/BCL2 ratio were significantly changed after CAP treatment in B16-F10 tumor cells in comparison to untreated controls (BAX (*P* = 0.028), BCL2 and CASP3 (*P* = 0.014), and BAX/BCL2 (*P* < 0.0001)). CUR significantly changed the mRNA expression of BCL2 and CASP3 in B16-F10 tumor cells in comparison to untreated control cells (BCL2 (*P* = 0.039) and CASP3 (*P* = 0.031)). However, the BAX gene did not significantly increase in the CUR-treated cells, and BAX/BCL2 ratio was significantly increased in B16-F10 tumor cells (*P* < 0.0001). The expression of BAX (*P* = 0.034), BCL2 (*P* = 0.042), and BAX/BCL2 ratio (*P* < 0.0001) but not CASP3 was significantly altered after combination therapy in B16-F10 cells in comparison with untreated cells. CAP and CUR treatments had no significant effects on the expression of apoptotic genes in L929 normal cells. However, the expression pattern of apoptotic genes in receiving the CAP and CUR combination L929 cells was slightly different. Simultaneous CAP and CUR treatment increased the expression pattern of CASP3 (*P* = 0.014) and BAX/BCL2 ratio (*P* < 0.0001), along with decreasing BCL2 (*P* = 0.027) but had no effect on BAX expression. The comparison of combination therapy with monotherapy with CAP or CUR revealed that CAP and CUR combination had no significant effect on apoptotic gene expression in comparison with CAP or CUR monotherapy.

### 3.7. Blocking of AQP-1 by CUR

**Figure 7:**
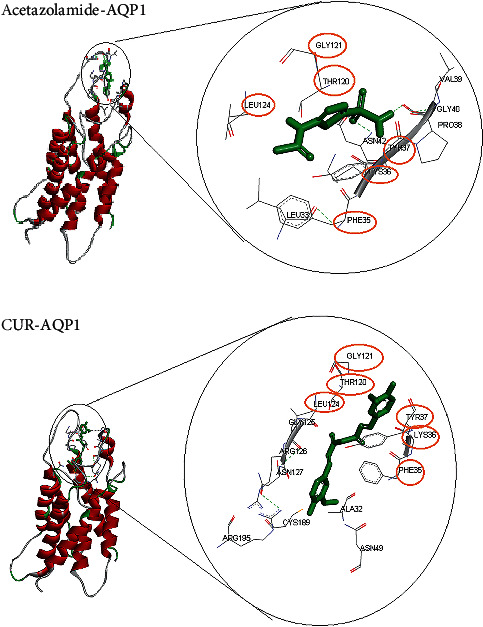
Comparison of interaction between AQP-1 with CUR and acetazolamide using AutoDock docking model Vina. To visualize interaction points and ligands more, interacting residues of the protein with ligands are shown and labeled. Two ligands (CUR and acetazolamide) interacted in the same interaction binding with the receptor. The docked model was visualized by Discovery Studio 4.5 software. CUR: curcumin; AQP1: aquaporin-1.

Analysis of docking was carried out between AQP-1, CUR, and acetazolamide separately. The lowest binding energy of each complex shows the highest binding affinity between the structures in the complex. The binding energy of CUR is near to the binding energy of acetazolamide (-6.3 and -4.5 kcal/mol, respectively). The docked complexes are shown in Figure 7. As it indicated, CUR and acetazolamide bind to AQP-1 in a similar location. Also, amino acids of GLY121, THR120, LEU124, PHE35, LYS36, and TYR34 were the same binding sites of CUR and acetazolamide.

## 4. Discussion

In the last few years, the combination therapy with CAP and other treatments has claimed that may promise cure of cancer [15, 29–32, 38–42]. This study revealed that CAP had more cytotoxic effect on tumor cells and did not have a toxic effect on normal cells. In addition, the results of the AO/EB staining and flow cytometry analysis confirmed that CAP induces significant apoptosis in the melanoma cancer cells but not in normal cells. Analysis of apoptosis-related genes, including BAX, BCL2, and CASP3, and also BAX/BCL2 ratio indicated CAP induces apoptotic pathway selectivity in tumor cells. Our results are in line with other studies that reported the effect of CAP on cytotoxicity and apoptosis in tumor cell lines [5, 6, 8, 40, 43–48]. Other studies proved evidences that CUR has a cytotoxic effect and induces apoptosis in cancer cell lines [49–56]. In this study, for the first time, we compared the effect of CUR on cytotoxicity and apoptosis of the B16-F10 cells in comparison with L929 normal cells. Our results showed CUR significantly decreased the viability of B16-F10 tumor cells and with a less intensity on L929 normal cells. Also, CUR activates apoptosis pathways in B16-F10 melanoma cells more than in L929 normal cells by increasing the expression of CASP3 and decreasing BCL2 expression. The combination therapy of CAP and CUR showed more toxic effect on tumor cells than the CUR treatment but not significant toxicity compared to the CAP monotherapy in 24 and 48 hours. AO/EB fluorescence staining and flow cytometry analysis also revealed that CAP and CUR each alone induced apoptosis and necrosis in B16-F10 melanoma cells. However, the combination therapy did not induce apoptosis in the cancer cells more than the monotherapy with CAP or CUR. Analysis of apoptosis-related genes, including BAX, BCL2, and CASP3 expression, suggested that the combination therapy did not have more effect in inducing apoptotic pathways in both cancer and normal cell lines in comparison with CAP or CUR alone. The BAX/BCL2 ratio demonstrates the stability and balance between the expression levels of pro- and antiapoptotic genes [57]. Our results showed the combination therapy increased BAX/BCL2 ratio in both tumor and normal cell lines than CAP or CUR treatments. In addition, all three treatment approaches showed a significant increase in the BAX/BCL2 ratio in B16-F10 cells in comparison with L929 cells. Therefore, it can be concluded that combination therapy does not have a better effect than treatment with any of CUR or CAP.

The inhibitory activity of CUR on aquaporin channels is a probable molecular mechanism for reducing the simultaneous effect of combination therapy on the apoptosis of the cancer cells. Previous studies indicated CUR has an inhibitory activity on aquaporin channels despite its ROS production and decreased entrance of reactive species into the cell via these channels [58–60]. The findings of docking modelling were in line with previous studies. Interaction of CUR with AQP-1 structure was the same in interacting location and intensity as interaction of acetazolamide, a carbonic anhydrase inhibitor of AQP-1 and AQP-4 [61–65], with this channel. In other words, like acetazolamide, curcumin inhibits the penetration of CAP-induced excess ROS into cancer cells by occupying AQP channels. Cancer cells express more AQPs in their cytoplasmic membrane than normal cells; thus, combination therapy of CAP and CUR has more effect on B16-F10 when compared to the L929 cell line. Previous studies reported the solvent can affect the physicochemical properties of CUR [66–68]. Therefore, DMSO and culture medium may have a role in the interactions between CUR and AQP channels. More investigations are required to prove this hypothesis. Other reasons that can justify this event include aquaporin channels are the most important ROS transporters into the cell; however, the rate of ROS transporter into the cell has a certain capacity [69, 70]. When B16-F10 cells are treated with a combination of CAP and CUR, the penetration of ROS in these cells is reduced, because these cells are saturated with ROS. Also, infiltration of high levels of ROS into the cells causes cellular oxidation and induction of necrosis instead of apoptosis [71–74], although CUR can inhibit melanoma cancer via other pathways [75]. Therefore, combination therapy has some cytotoxic effect on the cancer cells.

## 5. Conclusions

Our findings suggest CAP to selectively induce apoptosis in tumor cells. In addition, CUR induced apoptosis in cancer cells more than normal cells. Combination therapy with CUR and CAP induces cell death in cancer cells more than normal cells, but does not improve cytotoxicity and apoptosis in melanoma B16-F10 cancer cells compared with monotherapy with CAP or CUR.

## Figures and Tables

**Table 1:** Primer sequences used for stem-loop RT-PCR assays.

Accession number	Gene name	Primers 5′→3′
NM_007527.3	BAX	Specific forward primer: GCGGCTGCTTGTCTGGATC USI RT-PCR primer: GTCGTATCCAGTGCTGCGACCGTATGGATGTGTCTGCGGCGTTTTATCATGCACTGGATACGACCGGTGAGGACTC
NM_009741.5	BCL2	Specific forward primer: CTACGAGTGGGATGCTGGAGATG USI RT-PCR primer: GTCGTATCCAGTGCTGCGACCGTATGGATGTGTCTGCGGCGTTTTATCATGCACTGGATACGACGCTGGAAGGAGA
NM_001284409.1	CASP3	Specific forward primer: CTCTACAGCACCTGGTTACTATTCC USI RT-PCR primer: GTCGTATCCAGTGCTGCGACCGTATGGATGTGTCTGCGGCGTTTTATCATGCACTGGATACGACGTTGCCACCTTC
NM_001289726.1	GAPDH	Specific forward primer: TTGTCAAGCTCATTTCCTGGTATG USI RT-PCR primer: GTCGTATCCAGTGCTGCGACCGTATGGATGTGTCTGCGGCGTTTTATCATGCACTGGATACGACGGAGGCCATGTAG

## Data Availability

No data were used to support this study.

## Supplementary Materials

Supplementary Materials Supplementary Figure 1: cytotoxic effect of combination therapy with CAP and 25 micro molar of CUR on B16-F10 melanoma cancer and L929 normal cell lines. Supplementary Figure 2: cytotoxic effect of combination therapy with CAP and 15 micro molar of CUR on B16-F10 melanoma cancer and L929 normal cell lines.
