# Supplementary material for: Combined Effect of Cold Atmospheric Plasma and Curcumin in Melanoma Cancer
Source: Biomed Res Int. 2021 Nov 16;2021:1969863. doi: 10.1155/2021/1969863 (PMC8610675; doi:10.1155/2021/1969863)
Supplement: Supplementary Materials — Supplementary Figure 1: cytotoxic effect of combination therapy with CAP and 25 micro molar of CUR on B16-F10 melanoma cancer and L929 normal cell lines. Supplementary Figure 2: cytotoxic effect of combination therapy with CAP and 15 micro molar of CUR on B16-F10 melanoma cancer and L929 normal cell lines. [file 1969863.f1.pdf]

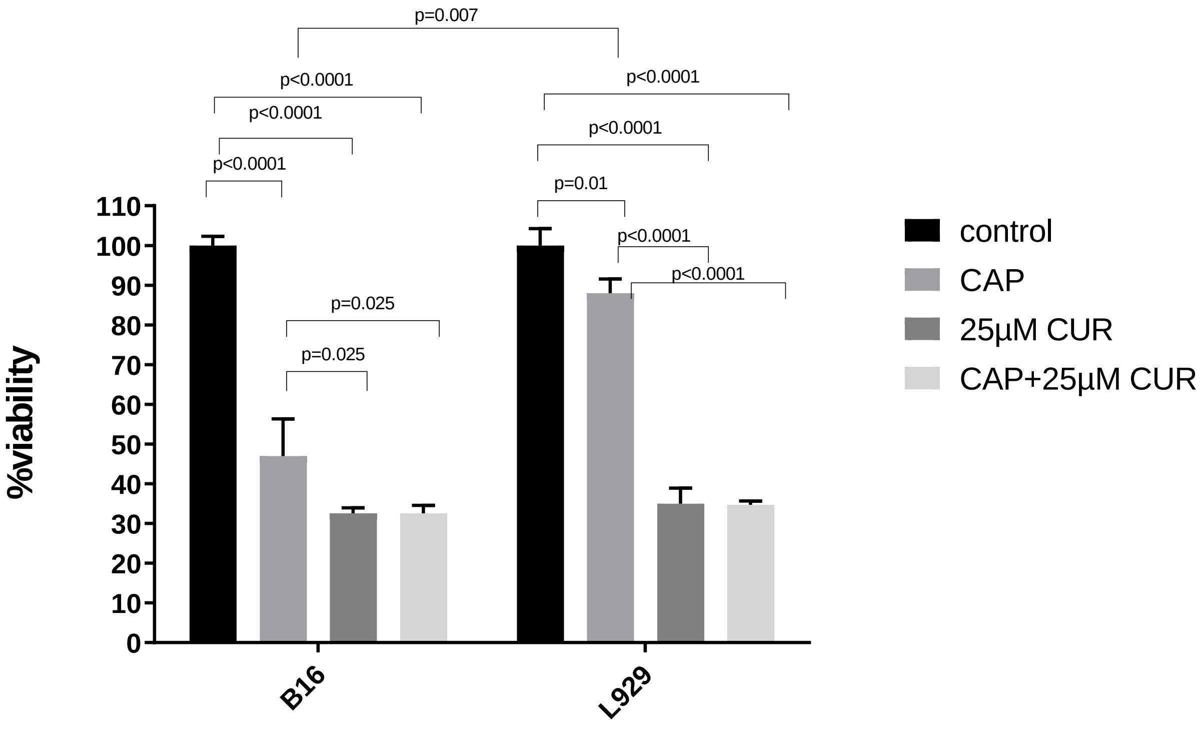

Supplementary Fig.1 Cytotoxic effect of combination therapy with CAP and 25 micro molar of CUR on B16-F10 melanoma cancer and L929 normal cell lines.

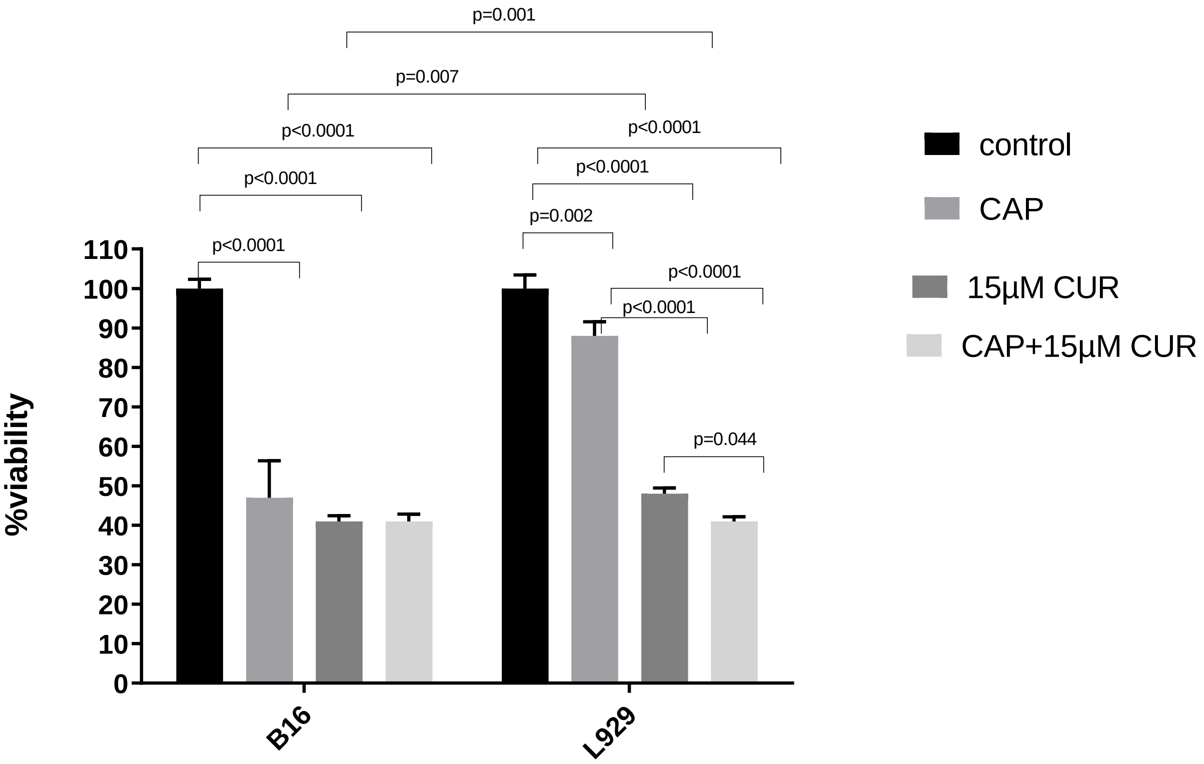

Supplementary Fig.2 Cytotoxic effect of combination therapy with CAP and 15 micro molar of CUR on B16-F10 melanoma cancer and L929 normal cell lines.
